# Novel Metabolomics Serum Biomarkers for Pancreatic Ductal Adenocarcinoma by the Comparison of Pre-, Postoperative and Normal Samples

**DOI:** 10.7150/jca.41250

**Published:** 2020-05-19

**Authors:** Xiaohan Zhang, Xiuyun Shi, Xin Lu, Yiqun Li, Chao Zhan, Muhammad Luqman Akhtar, Lijun Yang, Yunfan Bai, Jianxiang Zhao, Yu Wang, Yuanfei Yao, Yu Li, Huan Nie

**Affiliations:** 1 School of Life Science and Technology, Harbin Institute of Technology, Harbin, China.; 2 The Affiliated Tumor Hospital, Harbin Medical University, Harbin, China.

**Keywords:** Metabolomics, Pancreatic Ductal Adenocarcinoma, Multivariate analysis, Biomarkers

## Abstract

**Background:** Pancreatic ductal adenocarcinoma (PDAC) is one of the most aggressive human malignancies. The metabolomic approaches are developed to discover the novel biomarkers of PDAC.

**Methods:** 550 preoperative, postoperative PDAC and normal controls (NCs) serums were employed to characterize metabolic alterations in training and validation sets by LC-MS.

**Results:** The results of PLS-DA analysis indicated that three groups could be distinguished clearly and the post-PDAC group is adjacent to a normal group as compared with pre-PDAC group. Further results showed that histidinyl-lysine significantly increased whereas docosahexaenoic acid and LysoPC (14:0) decreased in pre-PDAC patients as compared with NCs. And these three markers had a significant tendency to recover after tumor resection. The validation set results revealed that for CA19-9 negative patients, 92.3% (12/13) of them can be screened using these three metabolites. The combination of these markers could significantly improve the diagnostic performance for PDAC, with higher sensitivity (0.93), specificity (0.92) and AUC (0.97). Moreover, network and pathways analyses explored the latent relationship among differential metabolites. The glycerolipid metabolism and primary bile acid synthesis showed variation in network and pathway analysis.

**Conclusions:** These three markers combined with CA199 displayed high sensitivity and specificity for detecting PDAC patients from NCs. The results indicated that these three metabolites could be regarded as potential biomarkers to distinguish PDAC from NCs.

## Introduction

It is estimated that the incidence of pancreatic ductal adenocarcinoma (PDAC) will be the second-leading cause of cancer-related deaths by 2030 due to the intractable detection and the poor prognoses 1,2. The 5-years survival rate of all PDAC patients has remained close to 5% 3-5. Clinical symptoms of PDAC patients are usually unremarkable in the early stage 6-7. More common clinical diagnostic methods for PDAC are mainly dependent on imaging examination and traditional protein biomarkers 8-9. Imaging examinations, such as magnetic resonance imaging, computed tomography, and endoscopic ultrasonography, have insufficient specificity and sensitivity for detecting PDAC in the early stage 10-12. On the other hand, the best-established serum biomarker is carbohydrate antigen 19-9 (CA19-9). Unfortunately, CA19-9 is not only insufficient for the early stages but also limited in the sensitivity (59%~64%) 13-15. So, at the time of diagnosis, only 20% of patients can remove their tumors, which could increase 5-years survival rate from 5% to 25%. Therefore, there is imperative to identify new biomarkers that could help in diagnosis of PDAC, which has become a medical emergency 16-17.

As an *omics* technology, metabolomics enable the global and untargeted measurement of small molecular (<1000Da) weight endogenous metabolites. It is a useful approach to understand known metabolic pathways and biological functional alterations in physiological and pathological responses 18. In recent years, metabolomics has been utilized to clarify the significant changes of tumor mechanisms and the discovery of new diagnostic biomarkers for early diagnosis 19. It is essential to distinguish the information between diseased and non-diseased status 20,21. Ultra-performance liquid chromatography-mass spectrometry has the most automated, reproducible and high-throughput characteristics, which is the most widely analytical platform for metabolomics 22. Thus, more and more research groups are taking advantage of metabolomics to the discovery of cancer biomarkers and understanding of pathophysiologic processes.

Numerous studies have reported to seek the multiple circulating metabolites signatures to discriminate pancreatic cancer cases from non-case controls 23-26, and pancreatitis cohorts in some researches 27-29. These selected individual biomarkers are available for the detection of PDAC and the receiver operating characteristic (ROC) of them have an outstanding result (AUC>0.9) 8,11,26,30,31. Although these screening modalities are generally able to detect PDAC; none of them have been implemented in daily practice so far due to poor consistency of results 32. Most of pancreatic cancer metabolomics studies used sample size ranging from 40 to 100 while only a few numbers of studies used more than 500 samples 33-35, and insufficient sample size may result in unrepresentative and variable results. Furthermore, there shall be a tendency to recover for effective biomarkers after tumor resection for the post-operative monitoring. This could be an excellent piece of evidence for whether or not it becomes a useful diagnosis marker. To date, however, no metabolomics study has investigated the relation between the resection of the tumor and the change of pancreatic cancer metabolism by comparing preoperative and postoperative serum samples 34.

In the present study, we have performed UPLC/Q-TOF MS based metabolite profiling analysis on 550 serum samples to screen out critical metabolite alterations that may discriminative biomolecules for PDAC diagnosis through utilizing preoperative and postoperative pathology in training cohorts. Combined with clinical information of the PDAC patients, three discriminative metabolites (Docosahexaenoic acid, LysoPC (14:0) and Histidinyl-Lysine) were determined to be independent predictors for PDAC diagnosis and its diagnostic performance was confirmed via independent validation analysis. The performance of three discriminative metabolites in PDAC was evaluated, and they provided a highly accurate classifier for delineating PDAC patients from NC with >97% accuracy (AUC = 0.97). Levels of three postoperative discriminative metabolites were closed to normal controls compared with paired preoperative PDAC group. In addition, correlation network and pathway analysis were carried out to understand the inter-relationship among discrepant metabolites. These results demonstrated the potential capability of the three metabolic biomarkers could be utilized to distinguish PDAC from NCs.

## Materials and Methods

### Sample Collection

550 serum samples from 431 populations were involved in this study including preoperative (pre-PDAC) and postoperative (post-PDAC) patients with PDAC and normal controls (NC). The training set consisted of pre-PDAC patients (n=185) and normal controls (n=146). Of the 185 pre-PDAC patients, 87 pairs of postoperative samples were collected. The validation set included another new PDAC preoperative samples (n=50), pairs of postoperative samples (n=32) and normal controls (n=50). All patients were diagnosed with pancreatic cancer for the first time and had no treatment before sampling, and were recruited and pathologically confirmed from the Affiliated Tumor Hospital at the Harbin Medical University; the serum samples from the healthy volunteers were obtained from the Fourth Affiliated Hospital of Harbin Medical University. Informed consents were obtained from all the enrolled participants before taking part in this study. The malignant severity was assessed by using the TNM classification system (the 8th edition of AJCC) and differentiation degree. The preoperative serum samples were obtained in the next morning after the patients were hospitalized and the postoperative ones were sampled in the morning on the seventh day after the operation when the pancreatic function was recovered. Control subjects were recruited on the basis that they had no history of cancer and the serum levels of the tumor markers CEA, CA19-9 and AFP were negative. Clinical information of all subjects was shown in Table 1. Notably, nearly half of the samples came from samples after surgery, leading to a high percentage of patients in stage I. And the proportion of patients with unknown staging information exceeds over 40% due to the mission of staging information for patients without surgery.

### Reagents and Chemicals

HPLC grade acetonitrile and methanol were purchased from Fisher Scientific (Waltham, MA, USA); formic acid (HPLC grade) was produced by Fluka (St. Louis, MO, USA); deionized water was provided by a Milli-Q ultrapure water system (Millipore, Billerica, USA).

### Sample Preparation

To provide a measurement of the stability and performance of the system, quality control samples (QCs) were prepared by pooling equal volume of supernatant of all samples in the identical corresponding dataset (i.e., training set or validation set). All serum supernatant were carefully collected with non-anticoagulant vacuum tubes and immediately centrifuged at 4000× g for 10 min at room temperature. The sample preparation was done according to the method described in our previous report 36. Serum samples were thawed on ice and 100 µl aliquots were mixed in 300 µl pre-cooled methanol/acetonitrile (1:1) for protein precipitation. Finally, the dried residue obtained after freeze-dried was re-dissolved by 100 µl of 50 % methanol.

### UPLC-Q/TOF MS Analysis

To ensure stability during analysis, samples were analyzed for quality control at the beginning and at the end of each running batch. According to the method described in a previous report, the injected sample volume 5 µl. Chromatographic separation was performed by the ultra-performance liquid chromatography (UPLC) system (Waters, Milford, USA) using a Waters BEH C18 column (2.1 mm × 100 mm, 1.7 µm) (Waters, Milford, MA) kept at 40 °C in ESI (+) and ESI (-). The elution flow rate was 0.30 mL/min to avoid insufficient nebulization. The optimized elution gradient was performed as follows: 0-0.5 min 1 % eluent A , 0.5-3.5 min 1-53 % eluent A , 3.5-7.5 min 53-70 % eluent A, 7.5-9 min 70-90 % eluent A , and then maintained at 9-13 min 90 % eluent A followed by alternating the gradient back to 13.1 to 15 min 1 % eluent A (0.1 % formic acid - acetonitrile (A) and 0.1 % formic acid - water (B)).

MS data identification and MS/MS acquisition were both performed in a dual electrospray ion source (Agilent, Santa Clara, CA, USA) with a 6520 series accurate quadrupole time-of-flight mass spectrometer (Q-TOF MS). MS data was collected in the positive and negative mode equipped with a scan rate of 1.5 spectra/s and the mass range was from 50 to 1100 m/z. The parameters for the acquisition were using the following settings: the capillary voltage, 4 and 3.5 kV in the positive and negative mode, respectively; the gas temperature, 330 °C; the flow rate, 10 L/min; the fragmentor, 100V; and the skimmer, 65 V.

### Statistical Analysis

Our initial analysis of the whole group was to process mass spectra data obtained by LC-MS using Qualitative Analysis B.04.00 to extract and align peaks. In the training set study, principle component analysis (PCA), partial least square discriminant analysis (PLS-DA) and orthogonal projection to latent structures discriminant analysis (OPLS-DA) were conducted to demonstrate that metabolomic profiling of PDAC patients and NC. 100 permutation tests of cross-validation were used to avoid over-fitting and to certify the credibility and stability of the PLS-DA and OPLS-DA models 37. Furthermore, the potential differential metabolites were selected via a univariate nonparametric Kruskal-Wallis rank sum test (p<0.05) and a multivariate random forest (RF) model (VIMP>1) was designed 38. To exclude the influence of gender, age and jaundice, the potential metabolites were selected through employing a multivariate logistic regression considering the three confounding factors. And the potential metabolites which were significant call-back in the paired postoperative serum samples were consequently used for the subsequent analysis. To obtain the discriminative metabolites with satisfactory predictive performances combined with CA19-9, we followed the criteria for discriminative metabolite determination from differential metabolites: (1) displaying a Pearson correlation coefficient (CC) with CA19-9 smaller than 0.15; (2) displaying a univariate AUC larger than 0.8 in the discrimination between PDAC and NC.

Hierarchical cluster analysis (HCA) was performed to visualize the significant intensity differences in the concentration levels of these differential metabolites in a heatmap. Subsequently, Pearson correlation analysis and multivariate logistic regression were applied to evaluate whether the differential metabolites were correlated with CA19-9 and the independent clinical factors and the PDAC diagnosis, respectively. Receiver operating characteristic (ROC) analysis was used to calculate the area under the ROC curve (AUC), sensitivity, and specificity values for the model to evaluate the predictive power of the discriminative metabolites alone and together with CA19-9 for PDAC diagnosis performance. The optimal cut-off value of the model was determined from its ROC curve. In the validation set study, the diagnostic model was evaluated using the AUC, sensitivity, specificity, and accuracy values observed at the cut-off value obtained in the training set study 39. Correlation network and a pathway analysis were performed to further illustrate the latent relationship between the differential metabolites in PDAC.

### Metabolite Identification and Screening

Identification of metabolites was completed as described in our previous work. Shortly, accurate mass measurements were subject to database searches in the public databases METLIN (http://metlin.scripps.edu/index.php). According to the RT, m/z and MS/MS spectrum of differential metabolites, they were well matched with those of authentic standards or confirmed spectrums in the public databases HMDB (http://www.hmdb.ca/), METLIN, as well as MassBank (http://www.massbank.jp/) 40.

## Results

### Metabolic Profiling of PDAC and NC

**Figure 1:**
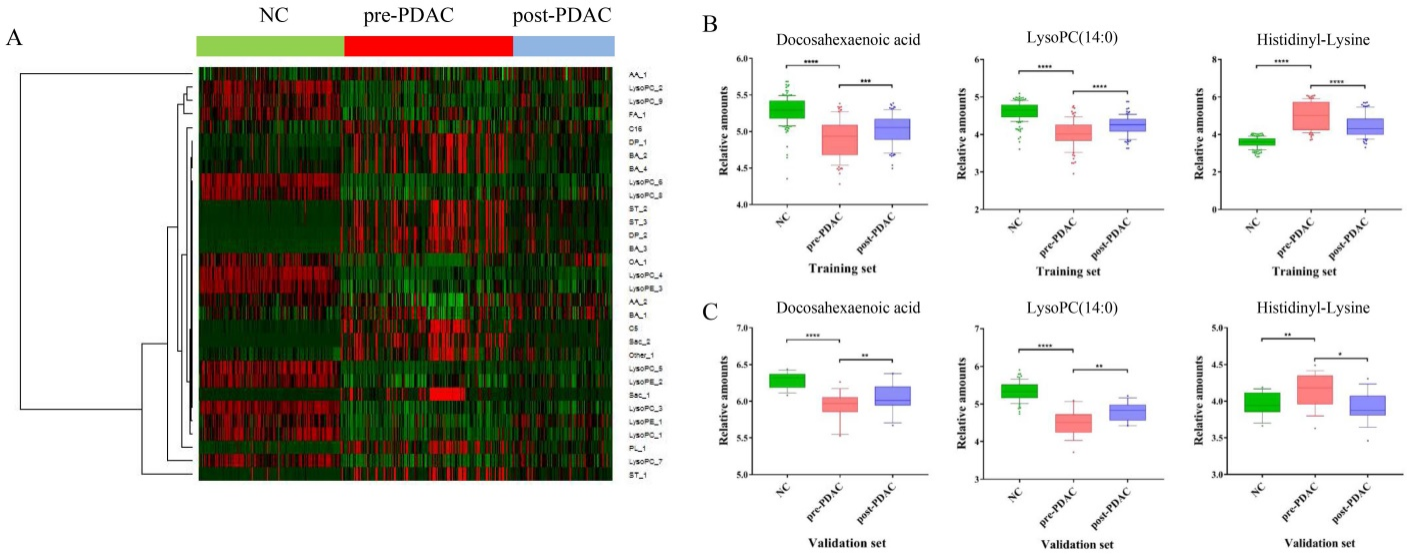
The workflow of the metabolomics data analysis.

**Figure 2:**
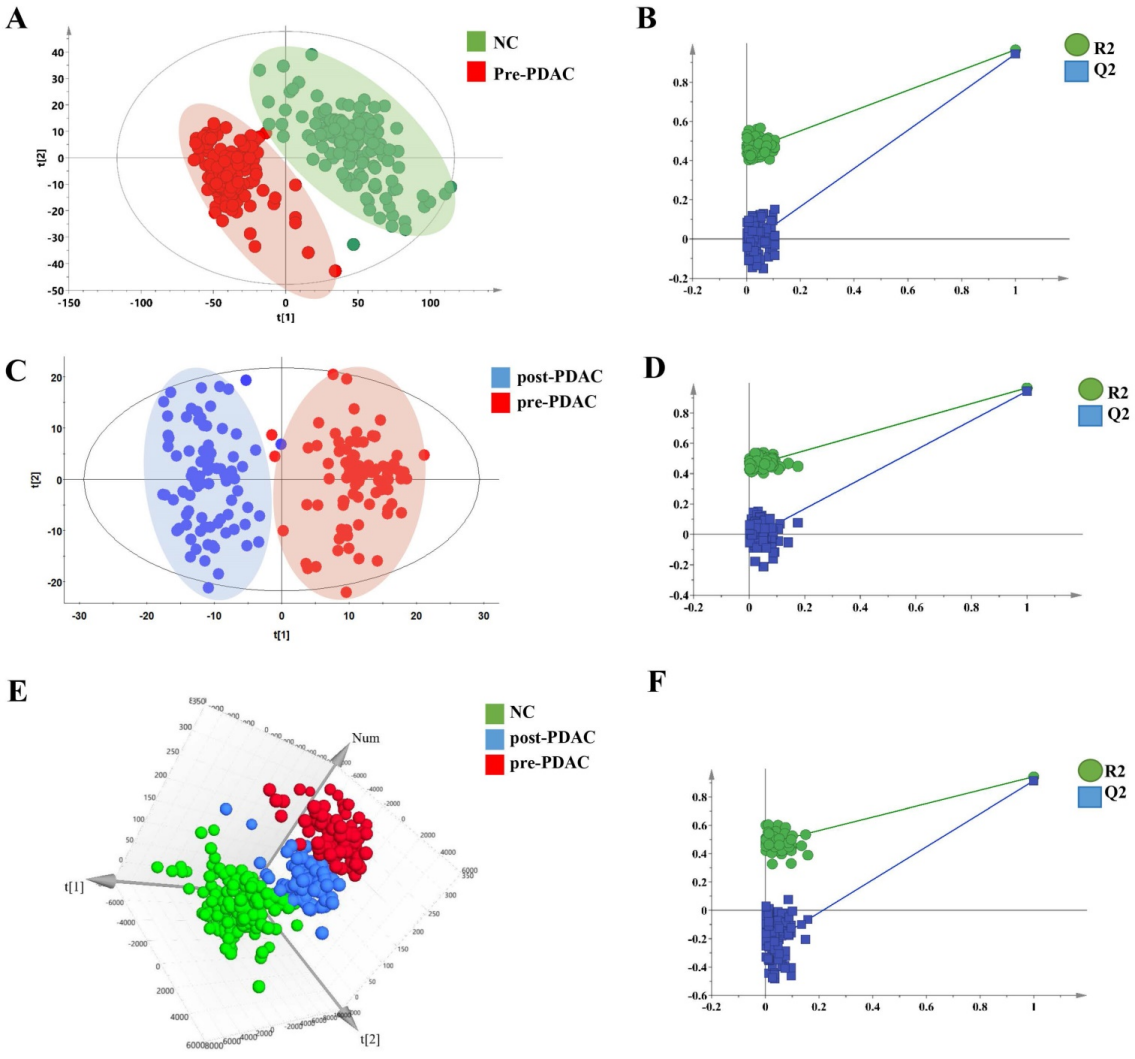
** Metabolic profiling analysis among NC, pre-PDAC and post-PDAC groups.** The score plot for PLS-DA (A) to discriminate pre-PDAC (n=185) and NC(n=146); and cross-validation plot obtained from 100 permutation tests (B); The score plot for OPLS-DA (C) to discriminate pair-wise pre-PDAC (n=87) and post-PDAC (n=87); and cross-validation plot obtained from 100 permutation tests (D); Three-dimensional score plot for PLS-DA (E) to discriminate pre-PDAC (n=87), post-PDAC (n=87) and NC (n=146); and cross-validation plot obtained from 100 permutation tests (F).

Metabolic profiling of PDAC and NC of 550 serum samples of the training set and validation set were acquired using UPLC/Q-TOF MS. The workflow for the metabolomics data analysis was presented in Figure 1. In training cohort, the typical basic peak chromatograms (BPC) of the pre-PDAC (n=185), post-PDAC (n=87) and NC (n=146) group in both the positive and negative ionization mode were shown in Figure S1. There was remarkable fluctuation of the height at the same arriving time of the chromatographic peaks of NC, pre-PDAC and post-PDAC group whether in positive and negative ionization mode. The PCA score plot for subjects in the training set was shown in Figure S2A, displaying a visible separation in the scores plots from patients with malignant disease and normal controls. Furthermore, the PLS-DA score plot (model parameters R2 = 0.88, Q2 = 0.87; Figure 2A) showed a clear separation of PDAC patients from NCs and no obvious over-fitting was observed in the permutation test (Figure 2B). These analyses indicated that there were obvious differences in the serum metabolic profiles between the PDAC and NC. Further, the OPLS-DA score plot described the differences of metabolic profiling in paired samples before and after surgery (Figure 2C). Then, the OPLS-DA score plot showed a clear separation of pre-PDAC patients from post-PDAC patients and no obvious over-fitting was observed in the permutation test (Figure 2D).

To seek for the metabolic changes caused by PDAC, we studied the effects of surgical resection on metabolomic profiles. The PCA (Figure S2B), three-dimensional PLS-DA (Figure 2E) and OPLS-DA (Figure S2C) pattern recognition techniques were applied to analyze the NC, pairing pre-PDAC and post-PDAC serum. The three-dimensional PLS-DA and OPLS-DA score plot for the subjects in the training set indicated that call-back postoperative samples could be separated from preoperative samples and normal samples (model parameters R2 = 0.68, Q2 = 0.64; R2=0.42, Q2=0.36), respectively. Notably, pre-PDCA patients were far away from the negative controls and postoperative patients, whereas the post-PDAC patients were located closely to NCs. It was evident that the good separation performance was achieved in PLS-DA and OPLS-DA model and the results of cross validation were reliable (Figure 2F and Figure S2D). These results revealed that the postoperative metabolic profiles had a tendency to recover after tumor resection.

Considering the influence of jaundice on the systemic metabolism, the PLS-DA model metabolic profiling analysis on NC, PDAC with and without jaundice groups were performed. The result showed that PDCA patients were far away from the negative controls. Notably, there was a certain tendency to separate PDAC with and without jaundice group (Figure S3). This result suggested that the jaundice might influence the selection of differential metabolites, and jaundice should be added as a confounding factor in screening differential metabolites.

### Selection and Identification of Differential Metabolites

On the basis of the metabolic profiling, pairwise comparisons of groups were carried out to further explore the differential metabolites responsible for the differences between pre-PDAC and NC. There were 8757 ions were found by LC-MS, from which there were 116 ions were selected as differential metabolites by Kruskal-Wallis rank sum test (p<0.05) and multivariate random forest (VIMP>1), which have been identified by MS-MS. In addition, to exclude the disordered metabolites caused by jaundice, gender, and age, the logistic regression was used to analyze the 116 differential metabolites. The results showed that 11 potential differential metabolites affected by jaundice, gender and age have been removed (Table S1), and it is proved that three of them has a relationship with jaundice or bilirubin by previous studies 41-43. Finally, the significant call-back metabolites were screened comparing the preoperative and postoperative PDAC pairs of samples, which were regarded as useful markers. 31 metabolites were found have significant call-back (the adjusted t-test's *p* value <0.05), including 17 negative ions mode (ESI-) and 14 positive ion mode (ESI+) (Table S2).

**Figure 3:**
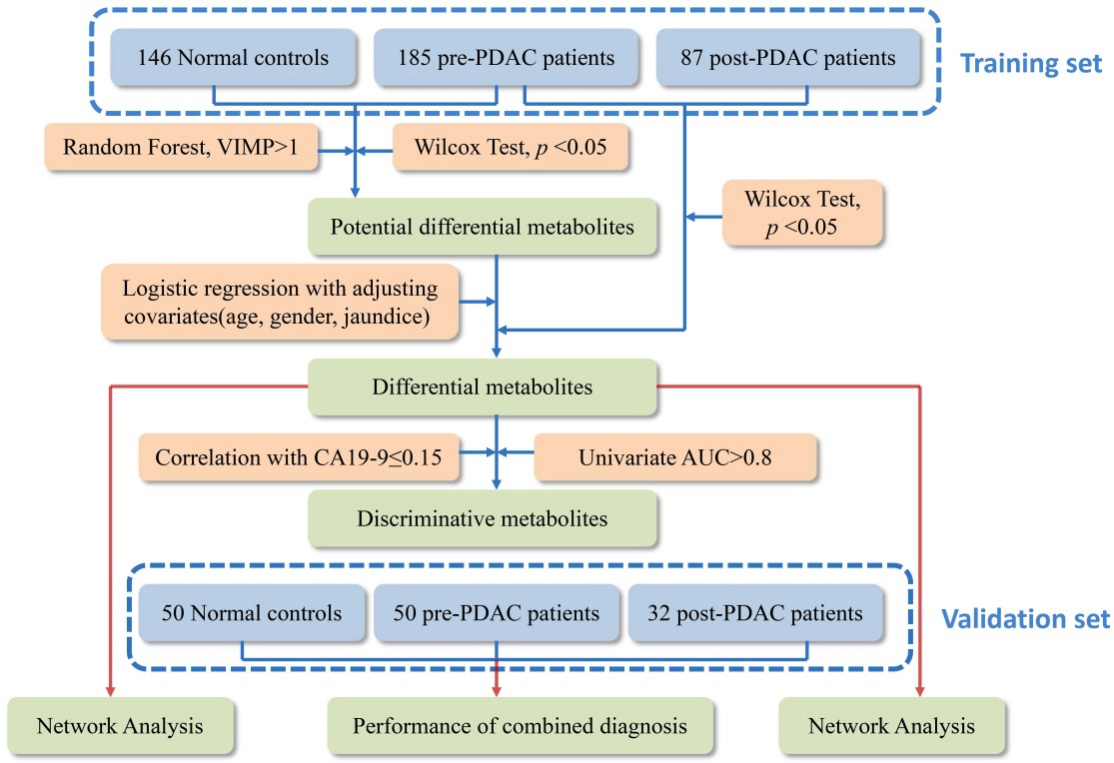
** HCA-heatmap and the expression of discriminative metabolites.** (A) HCA-heatmap plot indicating relative levels of differential metabolites in samples of the training set. (B) Box plots for comparing concentration levels of the three discriminative metabolites in different groups in the training set. (C) Box plots for comparing concentration levels of the three discriminative metabolites in different groups in validation set. * *p*< 0.05 ** *p*<0.01 *** *p*< 0.001 **** *p*< 0.0001.

To provide a different perspective into the group segregation, the HCA-heatmap for all the differential metabolites was presented in Figure 3A. In the HCA-heatmap diagram, the pre-PDAC group observations were completely separated from the post-PDAC and NC group. Overall, red cluster represented masses with mean elevation of 16 metabolites among PDAC patients, while green clusters represented masses with reduction of 15 metabolites. Additionally, the post-PDAC subjects were generally similar to NCs. This method was the same as the grouping patterns shown in the PLS-DA score plot.

### Diagnostic Performance and Verification of Discriminative Metabolites in External Validation Set

Three of these difference metabolites, docosahexaenoic acid (FA_1), LysoPC (14:0) (LysoPC_1), histidinyl-Lysine (DP_1) have been selected by a series of analysis processes (|CC|<0.15 and ACU > 0.8), and might be useful for PDAC diagnosis and prognosis. The external validation set, another batch of serum sample including NC (n=50), pre-PDAC (n=50) and post-PDAC cases (n=32), was collected and analyzed to validate the reliability of these three potential marker candidates. The same methods of sample pretreatment, instrumental detection, and data analysis were utilized. These three metabolites, docosahexaenoic acid, LysoPC (14:0) and histidinyl-Lysine showed significant differences (*p* < 0.05), and similar variable tendencies with those of the training set (Figure 3B and Figure 3C). Since the surgical approach (distal pancreatectomy and pancreaticoduodenectomy) has a totally different postoperative recovery, we examined the influence of surgical approach on three discriminative metabolites (Figure S4). The results showed that three discriminative metabolites were unaffected by surgical approach of patients.

**Figure 4:**
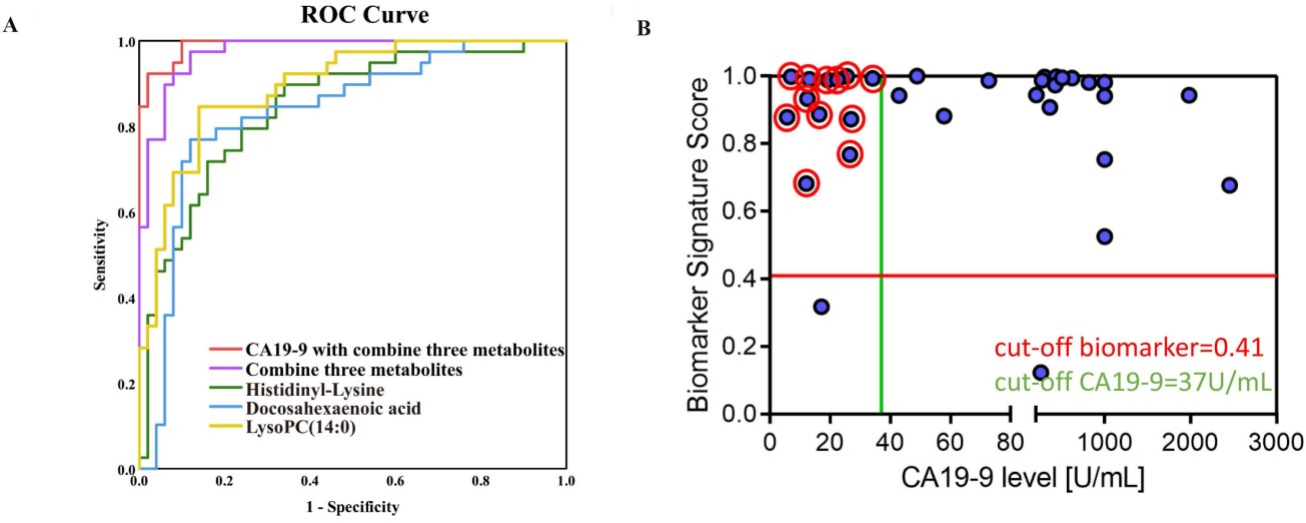
** Evaluation of potential biomarkers. Classiers are the biomarker signature generated in the training set and presented here for the validation set.** (A) Scatter plot for graphical representation of the biomarker signature score. Y-axis score of biomarker signature with the cut-off ≥ 0.41 and CA19-9 on the X axis with the cut-off ≥ 37 U/mL (< 37µg/mL as CA19-9-negative). (B) Blue circles are pancreatic cancer (n=34).

ROC curves of Docosahexaenoic acid, LysoPC (14:0) and Histidinyl-Lysine are shown in the validation set, respectively (Figure 4A). The sensitivity (Se), specificity (Sp), and area under the ROC curve were showed in Table S3 for three discriminative metabolites. Furthermore, three metabolites were defined as a combinational marker with favorable classification capability. The Se, Sp and AUC were 0.93, 0.92 and 0.97 for using this combinational marker to distinguish PDAC from NC. Further, the combinational markers with CA19-9 in the prediction of PDAC displayed a Se, Sp and AUC of 0.95, 0.98 and 0.99, respectively (Table S3). These AUC values indicated a satisfactory performance in the validation data sets, with remarkable sensitivity and specificity to accurately stratify subjects into correct groups. In addition, the dynamic changes in the normal controls and PDAC patients in different pathological stages (TNM staging system) were investigated (Figure S5A). The results showed that the level of Histidinyl-Lysine was related with progression of PDAC. Besides, Docosahexaenoic acid, LysoPC (14:0) and Histidinyl-Lysine showed an excellent performance (AUC>0.8) for stage I PDAC patients in training set (Figure S5B). These findings suggested that the discriminative metabolites might be useful for PDAC early diagnosis and prognosis.

Moreover, for the CA19-9-negative patients from the validation set, the combinational markers had a more ideal accuracy (Figure 4B). It was noteworthy that the CA19-9 value some of these cases were < 37 µg/mL, thus the CA19-9-negative patients cannot be distinguished by the serum CA19-9 level. 92.3% PDAC patients (12/13) who could not be screened by CA19-9 showed a positive result through the three candidate diagnostic metabolites. For one third (12/34) of the patients, our results would help to improve the diagnostic workup and treatment stratification. These results indicated that the discriminative metabolites could provide a comparable diagnostic performance of CA19-9 and the prediction of CA19-9-negative patients, which allow these metabolites potentially contribution to PDAC diagnosis in clinical practice.

Notably, after tumor resection, the postoperative serum level of LysoPC (14:0) inclined to normal level. Similarly, it was clear that most of the LysoPCs (LysoPC (15:0), LysoPC (P-16:0), LysoPC (17:0) and LysoPC (20:4(8Z,11Z,14Z,17Z)) were down-regulated in the preoperative conditions in comparison to healthy controls, and the elevation was obvious after resection of tumor in the training and validation set (Figure S6A and Figure S6B). Compared with pre-PDAC serum patients, post-PDAC patients have a tendency to return to normal. Due to tumor removal, the change of body metabolism made the level of LysoPC (14:0) and LysoPCs family call back.

### Correlation Network Analysis

**Figure 5:**
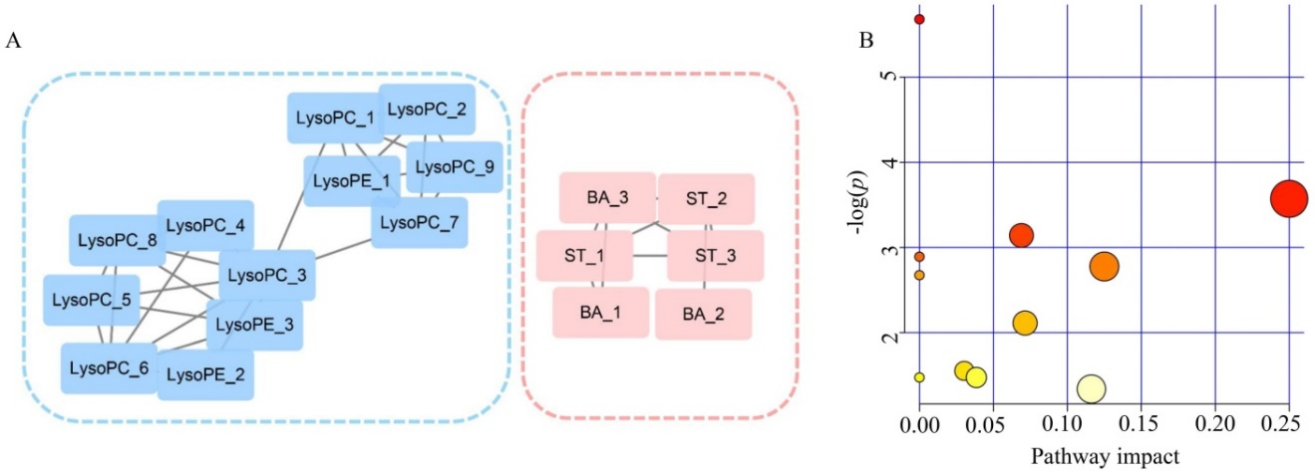
** Analyzed of the correlation network and pathways altered in PDAC.** (A) Correlation network constructed with 18 differential metabolites (Pearson correlation analysis, |r| > 0.6). Blue sub-network constructed with glycerophospholipids (LysoPCs and LysoPEs). Red sub-network constructed with steroids (ST) and bile acid (BA). Nodes in red and blue represent the metabolites down-regulated and up-regulated in PDAC, respectively. (B) Significantly changed pathways. Disordered pathways in PDAC group; small *p* value and big pathway impact factor indicate that the pathway is greatly influenced.

A correlation network was built on the basis of exploiting the latent relationships between the differential metabolites in PDAC, which ensured the robustness and reliability of the network construction. A total of 17 nodes and 26 edges were recruited in the network diagram in a circular layout on the criteria of a correlation coefficient ≥0.6 (Figure 5A). In accordance with the molecular composition and transforming relationship of metabolites of different classes, the entire network could be generally divided into two subnetworks. Glycerophospholipids (LysoPCs and LysoPEs) showed down-regulated concentration levels (blue nodes) in PDAC patients whereas steroids (ST) and bile acids (BA) were up-regulated (red nodes). The intra-category gathering landscapes could be clearly observed in metabolites of different classes in the network diagram, suggesting the underlying transformation of substances and energy in PDAC.

### Pathway Analysis

To further investigate the biochemical perturbation correlated with PDAC, an overview of the systematic metabolome changes on the basis of pathway analysis were conducted. PDCA-induced metabolic perturbation was analyzed from the perspective of pathway enrichment analysis combined with the topology analysis. The biological pathways involved in the metabolism of these 31 metabolites and their biological roles were determined by enrichment analysis using MetaboAnalyst (Figure 5B). A total of 10 matched metabolic pathways (Table S4) were shown according to *p* values from the pathway enrichment analysis (y-axis) and pathway impact values from pathway topology analysis (x-axis), the most impacted pathways colored in red. More attention should be paid to pathway with high impact values and pathway enrichment analysis (*p*<0.05). The perturbation of metabolism pathway includes phenylalanine, tyrosine and tryptophan biosynthesis, ubiquinone and other terpenoid-quinone biosynthesis and so on. These metabolic anomalies were found to be primarily involved in amino acid metabolism, lipid metabolism, and energy metabolism.

## Discussion

PDAC is burdened with a 5-year survival rate of around 5% and will be the second leading cause of cancer-related death by 2030 2. Therefore, it is necessary to improve the screening and diagnostic method for PDAC. Metabolomics, the 'omics technique' subject to environmental influences, has been proposed to be useful for identifying new biomarkers for PDAC early diagnosis 44. PDAC has a significant heterogeneity within the tumors of individuals 45,46. This calls for large sample sizes to ensure adequate representation of subtypes 47. Furthermore, biomarker development programmers required samples to be separated into independent training and validation sets 47. In our metabolomics analysis, this conventional route that benefits from a large population 46 was adopted in this study. And the samples were divided into training and validation sets, preoperative and postoperative, which guaranteed the reliability of the results. In order to select discriminative metabolites, we employed the strategy that was considered to be the influence of clinical factors (gender and age), which enhances the clinical reliability for epidemiological studies. Therefore, our metabolomics approach is acceptable as a screening method for large populations.

To screen out the discriminative metabolites that have satisfactory predictive performances alone or combined with CA19-9 from differential metabolites, the three criteria were followed in training set: (a) employing a Pearson correlation analysis to exclude the metabolites that have the correlation coefficient with CA19-9 greater than 0.15; (b) employing an area under the curve (AUC) value larger than 0.8. One important aspect of the data-modeling procedures lays in the predictive ability in terms of sensitivity (Se), specificity (Sp), and area under the ROC curve (AUC) in the external validation set distinguishing malignant pancreatic disease from normal controls. Previously metabolomics efforts have been made to compare PDAC and control samples. However, it is difficult to apply to the clinic because their models consist of many different metabolites and AUC of their model only maintained in 0.7~0.8 in external validation set 52, 53. Jiang and colleagues suggested TSGF as a candidate serum biomarker for pancreatic cancer and found that it displayed 91.6% sensitivity and 83% specificity 54. However, its sensitivity for early stages pancreatic cancer was decreased to 60.0~75.0%. In our study, to validate the reliability of these three potential marker candidates, we collected and analyzed another batch of serum sample including NC cases (n=50) and PDAC cases (n=50). The same methods of sample pretreatment, instrumental detection, and data analysis were utilized. Our diagnostic performance sensitivity (0.93), specificity (0.92) and AUC (0.97) of combinational marker were much more enhanced than CA19-9. Combinational markers performed an accuracy of 92.3% for CA19-9 negative patients (12/13), which provide a complement to the analysis for unsatisfactory performance of CA19-9. The combinational markers with CA19-9 in the prediction of PDAC displayed a Se, Sp and AUC of 0.95, 0.98 and 0.99, respectively. Indeed, our combinational markers effectively assist the diagnostic performance of CA19-9.

For the discriminative metabolites, docosahexaenoic acid and LysoPC (14:0) were down-regulated, while histidinyl-lysine was up-regulated in PDAC patients. As the complex structure of LysoPCs, UPLC-MS is the best way to determine accurately the levels of each individual LysoPCs from minimal amounts of serum. LysoPCs are a class of chemical compounds that are derived from PC 48. In our study, not only LysoPC (14:0) but also the other members of the LysoPCs family (LysoPC (15:0), LysoPC (P-16:0), LysoPC (17:0) and LysoPC (20:4)) were down-regulated in PDAC. It is revealed that the LysoPCs might relate with the carcinogenesis and progression of PDAC. The long chain dietary polyunsaturated fatty acid have been found to enhance various cellular responses that reduce cancer cell viability and decrease proliferation both in vitro and in vivo 49-51. A decrease in docosahexaenoic acid indicates a disorder of fatty acids. In addition, a dipeptide is an organic compound derived from two amino acids which can identical different. Although dipeptides were generally considered as incomplete breakdown products of protein digestion or protein catabolism, the specific metabolic mechanism of dipeptides in PDAC patients' remains rarely reported. It indicated that the amino acid metabolism was disordered and may bring disturbance of body metabolism in PDAC.

Due to alterations in the tumor cell and systemic metabolism, PDAC causes changes in circulating metabolites, which is central to the biology of PDAC 55. To capture the differential metabolites relationships in global changes, network analyses have been widely applied in metabolomics studies 56. In this study, phenylalanine, tyrosine and tryptophan biosynthesis, ubiquinone and other terpenoid-quinone biosynthesis were the majority of perturbed metabolic pathways.

In conclusion, our study showed that the discriminative metabolite selection strategy can readily and effectively be applied to serum metabolomics on the basis of a multivariate analysis. The selected diagnostic metabolites not only have the ability to diagnose PDAC from NCs, but also can effectively improve the diagnostic performance of CA19-9. All of diagnostic metabolites had a tendency to recur in postoperative samples, which suggests that the perturbation is coming from the tumors. Moreover, the correlation network and pathway analysis presented the relationships between discriminative metabolites and the disturbed biological mechanism in PDAC's development. These results will not only provide the potential for the improvement in diagnostic accuracy, but also the identification of altered metabolic pathways between PDAC and NCs, which may help us to understand the mechanisms of PDAC.

## Supplementary Material

Supplementary figures and tables.

## Figures and Tables

**Table 1:** Pathological and clinical characteristics of subjects in training set and validation set

Characteristics		Training set			Validation set		
		NC (n=146)	PDAC (n=185)	post-PDAC (n = 87)	NC (n=50)	PDAC (n=50)	post-PDAC (n = 32)
Age	≤40	17	24	2	1	3	3
	41~50	41	29	18	5	5	10
	51~60	52	63	30	19	19	8
	≥61	36	69	37	25	25	11
Sex	male	88	109	52	25	25	11
	female	58	76	35	25	25	21
Diabetes	yes	0	14	7	0	5	2
	no	146	130	53	50	37	28
	unknown	0	41	27	0	8	2
Hepatitis B	yes	0	7	4	0	2	0
	no	146	138	65	50	33	21
	unknown	0	40	18	0	15	11
TNM stage	I	—	85	69	—	28	17
	II~IV	—	28	7	—	14	9
	unknown	—	72	11	—	8	6
Differentiation	poor	—	55	28	—	11	10
	moderate	—	42	26	—	11	4
	high	—	41	30	—	19	13
	unknown	—	47	4	—	8	5
Jaundice	yes	—	65	32	—	12	7
	no	—	85	38	—	25	16
	unknown	—	35	17	—	13	9
Surgical	method 1	—	—	22	—	—	14
approach	method 2	—	—	40	—	—	8
	unknown	—	—	25	—	—	10
CA19-9	≤37	—	34	22	—	13	8
	>37	—	95	58	—	21	13
	unknown	—	56	7	—	16	11

Method 1: pancreatectomy; Method 2 pancreaticoduodenectomy.

## Abbreviations

UPLC: ultra-performance liquid chromatography
MS: mass spectrometry
TOF: time-of-flight
NMR: nuclear magnetic resonance spectroscopy
PDAC: pancreatic ductal adenocarcinoma
CA19-9: carbohydrate antigen 19-9
ROC: receiver operating characteristic
AUC: area under the curve
PCA: principle component analysis
HCA: hierarchical cluster analysis
PLS-DA: partial least square discriminant analysis
RF: random forest
QC: quality control
RT: retention time
m/z: mass-to-charge ratio
BPC: basic peak chromatogram
TNM: tumor node metastasis
Se: sensitivity
Sp: specificity
AA: amino acid
DP: dipeptide
LysoPC: lysophosphatidylcholine
PC: phosphatidylcholine
PE: phosphatidylethanolamine
FA: fatty acid
ST: sterol lipid
LysoPE: lysophosphatidylethanolamine
