# Supplementary material for: Novel Metabolomics Serum Biomarkers for Pancreatic Ductal Adenocarcinoma by the Comparison of Pre-, Postoperative and Normal Samples
Source: J Cancer. 2020 May 19;11(16):4641–51. doi: 10.7150/jca.41250 (PMC7330680; doi:10.7150/jca.41250)
Supplement: Supplementary file 1 — Supplementary figures and tables. [file jcav11p4641s1.pdf]

Novel Metabolomics Serum Biomarkers for Pancreatic Ductal Adenocarcinoma by the Comparison of Pre-, Postoperative and Normal Samples

Xiaohan Zhang[1], Xiuyun Shi[1], Xin Lu[1], Yiqun Li[1], Chao Zhan[2], Muhammad Luqman Akhtar[1], Lijun Yang[1], Yunfan Bai[1], Jianxiang Zhao[1], Yu Wang[1], Yuanfei Yao[2], Yu Li[*1] and Huan Nie[*1]

1.School of Life Science and Technology, Harbin Institute of Technology, Harbin, China

2.The Affiliated Tumor Hospital, Harbin Medical University, Harbin, China

***Corresponding author:**

Yu Li, Room 310, Building 2E, Science Park of Harbin Institute of Technology, No. 2 Yikuang Street, Nangang District, Harbin 150001, China; Fax: 86-451-86402691; Email: liyugene@hit.edu.cn

Huan Nie, Room 310, Building 2E, Science Park of Harbin Institute of Technology, No. 2 Yikuang Street, Nangang District, Harbin 150001, China; Fax: 86-0451-86402690; Email:nh1212@hit.edu.cn

Table S1 Summary of the metabolites affected by confounding factor

ID	Metabolites Name	*p* value
1	Succinic acid	0.158983
2	Tetradecanoylcarnitine	0.175737
3	Hydroxytetradecanoylcarnitine	0.176052
4	Propionyl-L-carnitine	0.194848
5	L-Threonine	0.280355
6	Creatine	0.388908
7	L-Palmitoylcarnitine	0.519071
8	L-Lysine	0.575777
9	L-Leucine	0.613617
10	Uric acid	0.800489
11	Phenylalanylphenylalanine	0.158983

Table S2 Summary of the identified in serum metabolic profiling

ID	Selected Metabolites	Measured (mz)	Retention time(min)	Formula	Ion mode	Class	VIMP	Trend
1	L-Methionine	150.0579	1.76	$C_5H_{11}NO_2$	M+H	AA_1	1.35	↑ *
2	Glycocholic Acid	504.2732	4.99	$C_{26}H_{43}NO_6$	M+K	BA_1	1.89	↑ **
3	Glycochenodeoxycholate-3-sulfate	528.2647	4.95	$C_{26}H_{43}NO_9S$	M-H	BA_2	1.41	↑ **
4	Tauroursocholic acid	539.2833	4.7	$C_{26}H_{45}NO_7S$	M+H+Na	BA_3	1.35	↑ **
5	4-Methoxybenzyl butanoate	207.102	5.37	$C_{12}H_{16}O_3$	M-H	BA_4	4.31	↑ **
6	Valerylcarnitine	246.1686	3.48	$C_{12}H_{23}NO_4$	M+H	C5	2.13	↑ **
7	Hexadecanedioic acid mono-L-carnitine ester	430.3116	5.16	$C_{23}H_{43}NO_6$	M+H	C16	3.48	↑ **
8	Histidinyl-Lysine	328.1623	6.02	$C_{12}H_{21}N_5O_3$	M+FA-H	DP_1	3.39	↑ **
9	Glutamylglutamic acid	273.0799	4.44	$C_10H_{14}N_2O_7$	M-H	DP_2	5.33	↑ **
10	(2S,4R)-4-(9H-Pyrido[3,4-b] indol-1-yl)-1,2,4-butanetriol	317.1146	4.05	$C_{15}H_{16}N_2O_3$	M+FA-H	Other_1	1.84	↑ **
11	Nepetaside	347.1603	4.87	$C_{16}H_{26}O_8$	M+H	PL_1	1.34	↑ **
12	1-[(5-Amino-5-carboxypentyl)amino]-1-d eoxyfructose	353.1611	4.93	$C_{12}H_{24}N_2O_7$	M+FA-H	Sac_1	2.15	↑ *
13	Bilirubin glucuronide	759.2882	4.55	$C_{39}H_{44}N_4O_{12}$	M-H	Sac_2	1	↑ **
14	Glycocholate	466.3169	4.98	$C_{26}H_{43}NO_6$	M+H	ST_1	1	↑ **
15	Taurochenodeoxycholate	498.2892	5.14	$C_{26}H_{45}NO_6S$	M-H	ST_2	1.22	↑ **
16	Taurocholate	514.2847	4.71	$C_{26}H_{45}NO_7S$	M-H	ST_3	1.34	↑ **
17	L-Tyrosine	180.0663	2.52	$C_9H_{11}NO_3$	M-H	AA_2	1.79	↓ **
18	Docosahexaenoic acid	327.2332	10.71	$C_{22}H_{32}O_2$	M-H	FA_1	1.36	↓ **
19	LysoPC(14:0)	468.3093	6.51	$C_{22}H_{46}NO_7P$	M+H	LysoPC_1	2.71	↓ **
20	LysoPC(15:0)	482.3253	7.51	$C_{23}H_{48}NO_7P$	M+H	LysoPC_2	2.07	↓ **
21	LysoPC(16:0)	540.3326	8.03	$C_{24}H_{50}NO_7P$	M+H	LysoPC_3	4.81	↓ **
22	LysoPC(P-16:0)	524.3362	8.87	$C_{24}H_{50}NO_6P$	M+FA-H	LysoPC_4	1.37	↓ **

23	LysoPC(17:0)	554.3466	8.96	$C_{25}H_{52}NO_{7}P$	M+FA-H	LysoPC_5	4.93	↓ **
24	LysoPC(18:0)	568.3633	9.82	$C_{26}H_{54}NO_{7}P$	M+H	LysoPC_6	2.25	↓ **
25	LysoPC(20:3(5Z,8Z,11Z))	546.3568	8.27	$C_{28}H_{52}NO_{7}P$	M+H	LysoPC_7	7.48	↓ **
26	LysoPC(20:4(8Z,11Z,14Z,17Z))	588.3317	7.67	$C_{28}H_{5}0NO_{7}P$	M+FA-H	LysoPC_8	7.44	↓ **
27	LysoPC(20:5(5Z,8Z,11Z,14Z,17Z))	542.3249	6.85	$C_{28}H_{48}NO_{7}P$	M+H	LysoPC_9	2.61	↓ **
28	LysoPE(0:0/20:5(5Z,8Z,11Z,14Z,17Z))	500.278	6.84	$C_{25}H_{42}NO_{7}P$	M+H	LysoPE_1	1.37	↓ **
29	LysoPE(18:0/0:0)	480.3101	9.77	$C_{23}H_{48}NO_{7}P$	M-H	LysoPE_2	1.11	↓ **
30	LysoPE(20:2(11Z,14Z)/0:0)	504.3098	7.69	$C_{25}H_{48}NO_{7}P$	M-H	LysoPE_3	1.35	↓ **
31	Indoxyl sulfate	212.0022	3.58	$C_{8}H_{7}NO_{4}S$	M-H	OA_1	1.00	↓ **

* $p < 0.05$; ** $p < 0.0001$, ↑ up-regulated in PDAC patients compared with NCs; ↓ down-regulated in PDAC patients compared with NCs

Table S3 Sensitivities, specificities and AUC values of Docosahexaenoic acid, LysoPC(14:0), Histidinyl-Lysine, combinations of discriminative metabolites and those with CA19-9

Case	Sensitivity	Specificity	AUC
Docosahexaenoic acid	0.88	0.77	0.83
LysoPC(14:0)	0.86	0.85	0.90
Histidinyl-Lysine	0.72	0.84	0.85
Ca19-9	0.73	0.94	0.85
Combination of discriminative metabolites	0.93	0.92	0.97
Combination of discriminative metabolites and CA19-9	0.95	0.98	0.99

Table S4 Summary of the significant metabolic pathways

ID	Pathway Name	*p*	Impact
1	Phenylalanine, tyrosine and tryptophan biosynthesis	7.61e4	0.10
2	Ubiquinone and other terpenoid-quinone biosynthesis	5.16e3	0.11
3	Tyrosine metabolism	1.59e4	0.08
4	Pentose and glucuronate interconversions	0.01	0.07
5	Aminoacyl-tRNA biosynthesis	0.01	0.06
6	Glycerophospholipid metabolism	0.06	0.08
7	Cysteine and methionine metabolism	0.08	0.00
8	Taurine and hypotaurine metabolism	0.09	0.03
9	Phenylalanine metabolism	0.09	0.25
10	Biosynthesis of unsaturated fatty acids	0.10	0.03

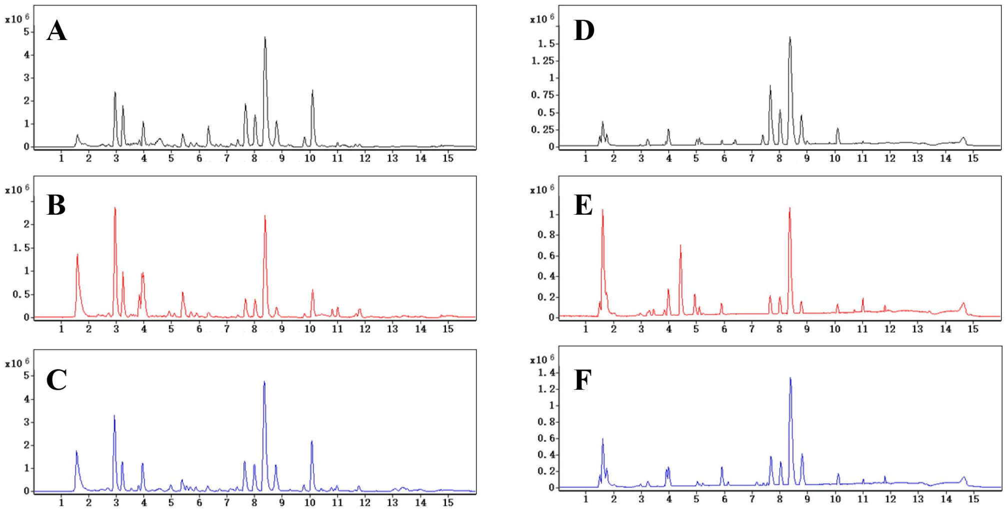

Figure S1 The typical UPLC/Q-TOF MS chromatograms for NC, pre-PDAC and post-PDAC group in both positive ion mode(A, B and C for NC, pre-PDAC and post-PDAC, respectively) and negative ion mode (D, E and F for NC, pre-PDAC and post-PDAC, respectively).

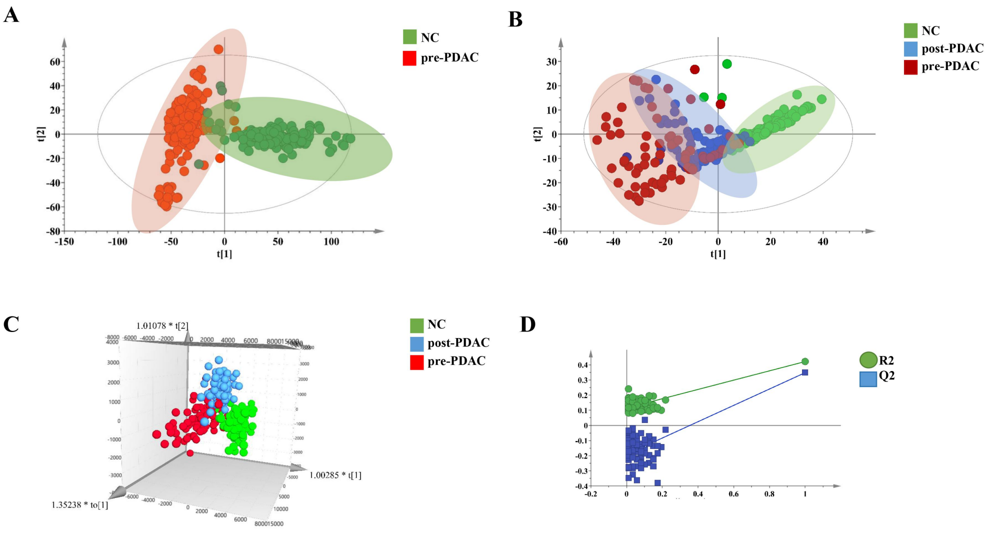

Figure S2 Metabolic profile analysis among NC, pre-PDAC and post-PDAC. The score plot for PCA (A) to discriminate pre-PDAC (n=185) and NC (n=146); The score plot for PCA (B) to discriminate pre-PDAC (n=87), post-PDAC(n=87) and NC (n=146); Three-dimensional score plot for OPLS-DA (C) to discriminate pre-PDAC (n=87), post-PDAC (n=87) and NC (n=146); and cross-validation plot obtained from 100 permutation tests (D).

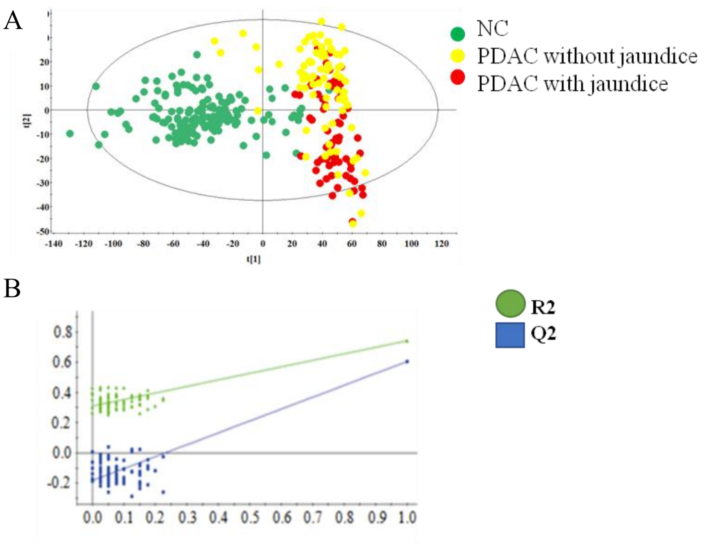

Figure S3 The score plot for PLS-DA and cross-validation. (A)The score plot for PLS-DA to discriminate PDAC with jaundice (n=65), PDAC without jaundice (n=85) and NC (n=146); (B) Cross-validation plot obtained from 100 permutation tests;

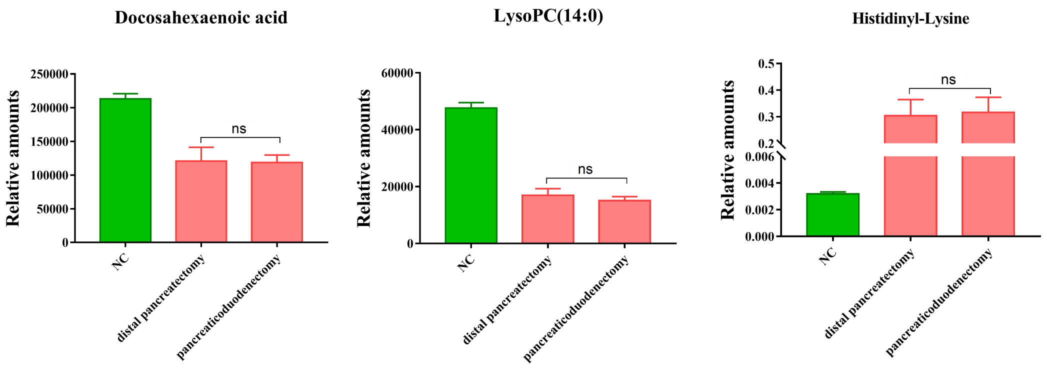

Figure S4 Box plots for comparing concentration levels of the three discriminative metabolites in NCs, distal pancreatectomy and pancreaticoduodenectomy groups in the training set.

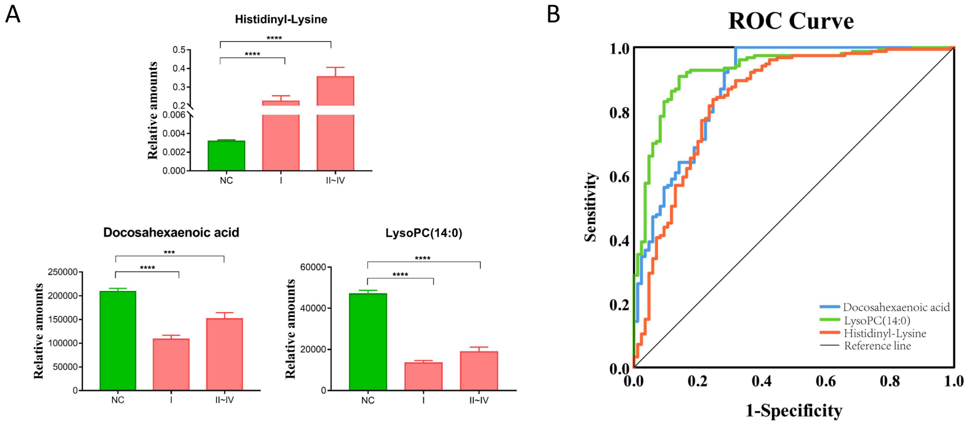

Figure S5 Three discriminative metabolites the dynamic changes in the normal controls and PDAC patients in different pathological stages. (A) Box plots for Docosahexaenoic acid, LysoPC(14:0), Histidinyl-Lysine in training set. (B) ROC curve of Docosahexaenoic acid, LysoPC(14:0), Histidinyl-Lysine, and to distinguish to PDAC from NC in training set.

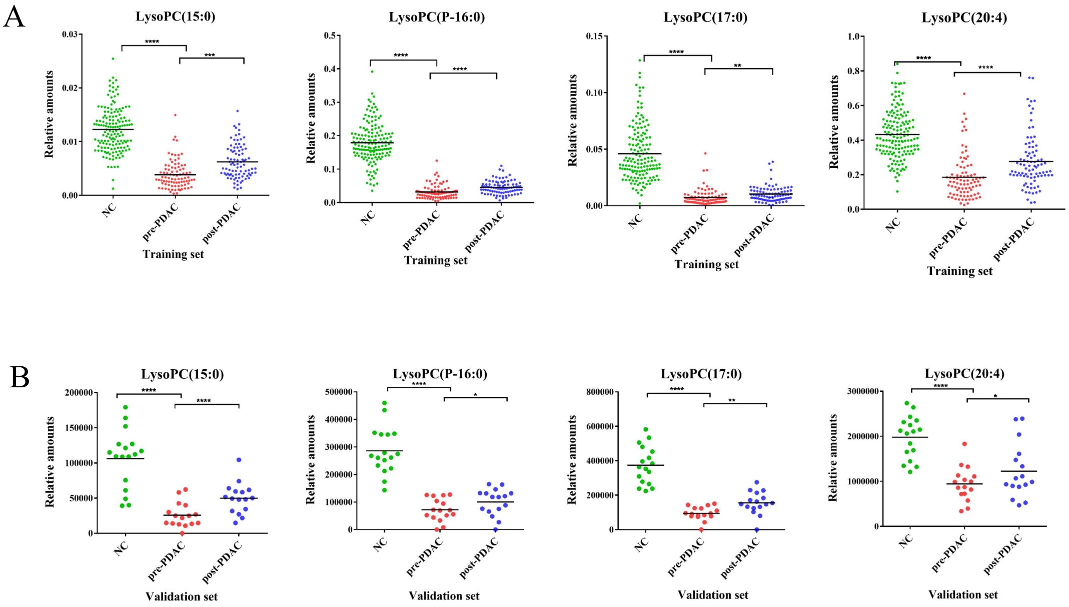

Figure S6 Metabolic changes among NC, per-PDAC and post-PDAC of differential LysoPC family. Box plots for LysoPC(15:0), LysoPC(P-16:0), LysoPC(17:0), LysoPC(20:4) in the training set(A) and validation set(B).
